# Characteristics and Circumstances Associated with Work-Related Suicides from the National Violent Death Reporting System, 2013–2017

**DOI:** 10.3390/ijerph18189538

**Published:** 2021-09-10

**Authors:** Corinne Peek-Asa, Ling Zhang, Cara Hamann, Jonathan Davis, Laura Schwab-Reese

**Affiliations:** 1 Injury Prevention Research Center, College of Public Health, University of Iowa, Iowa City, IA 52242, USA; ling-zhang-1@uiowa.edu (L.Z.); cara-hamann@uiowa.edu (C.H.); jonathan-davis@uiowa.edu (J.D.); 2 Department of Occupational and Environmental Health, College of Public Health, University of Iowa, Iowa City, IA 52242, USA; 3 Department of Biostatistics, College of Public Health, University of Iowa, Iowa City, IA 52242, USA; 4 Department of Epidemiology, College of Public Health, University of Iowa, Iowa City, IA 52242, USA; 5 Department of Public Health, College of Health and Human Sciences, Purdue University, West Lafeyette, IN 47905, USA; lschwabr@purdue.edu

**Keywords:** suicide, work-related suicide, occupational health

## Abstract

Workplaces are critical in suicide prevention because work-related factors can be associated with suicide, and because workplaces can be effective suicide prevention sites. Understanding the circumstances associated with work-related suicides can advance worksite prevention efforts. Data from the United States Centers for Disease Control and Prevention, National Violent Death Reporting System from 2013 to 2017 were used to examine characteristics and circumstances associated with work compared with non-work suicides. Work-related suicides included those indicated as work-related on the death certificate or in which the death investigation mentioned a work problem or work crisis. Of the 84,389 suicides, 12.1% had some relation to the decedent’s work. Males, those aged 21–54, and with at least a college education, were most likely to have work-related suicides. The circumstances most strongly associated with work-related suicide were financial problems (Odds Ratio (OR) = 4.7; 95% Confidence Interval (CI) = 4.5–5.0), prior depressed mood (OR = 2.4; 95% CI = 2.3–2.5), and eviction/loss of home (OR = 1.6; 95% CI = 1.4–1.7). Suicides among healthcare practitioners and management occupations had the highest odds of being work-related. Workplace wellness programs can consider incorporating services, such as financial planning and mental health services, as potentially up-stream approaches to prevent work-related suicide.

## 1. Introduction

In 2015, Trust for America’s Health issued a report called *Pain in the Nation*, which identified an “epidemic of despair” measured by increased deaths from suicide, substance use, and alcohol [1]. An update to the original report found that more than 156,000 Americans died from these causes in 2019, which was more than twice the number in 1999 [2]. Suicide rates in the United States are increasing at a rate of 2–5% per year, with increases experienced across nearly all sociodemographic groups [1,2,3]. Suicides are also increasing as a cause of occupational fatality. Since the Bureau of Labor Statistics began tracking workplace suicides in 1992, the number of workplace suicides has increased from 205 to 304 in 2018, a 48% increase [4,5], and the proportion of workplace fatalities that were suicides increased from 3% to 6% [6]. The relevance of these trends is highlighted in the media, including articles focused on workplace suicides in The Washington Post (9 January 2020) and Forbes (5 September 2020) [7,8].

The Census of Fatal Occupational Injuries, which relies on the death certificate, worker’s compensation records, and newspaper reports, estimates that 1% to 3% of suicides are work-related. Although the Census of Fatal Occupational Injuries has been the most comprehensive data to identify occupational fatalities, its sources have limited information on suicide circumstances and thus estimates for suicide are considerably low [9,10,11]. Data from the National Violent Death Reporting System, which includes more in-depth investigation of circumstances associated with suicide and homicide, identified work factors in 13.5% of suicides [12].

A growing evidence base focused on suicidality continues to identify myriad physical health, mental health, socioeconomic, and environmental risk factors associated with suicidal behavior [13,14,15,16,17]. Occupation and factors associated with work are emerging as recognized areas of suicide risk. For example, occupations such as military service, healthcare, mining, farming, and construction have been identified as having high suicide rates [18,19,20,21]. Work stress, job loss, and job-related financial stress have been identified as suicide risk factors [22,23,24]. However, most studies of work-related factors in suicide have either focused on overall prevalence, or have examined only individual risk factors (e.g., work stress) or individual sectors (e.g., military). Few studies have examined multiple work-related circumstances including all occupations.

Curtailing the increasing suicide trend will require an understanding of the circumstances related to suicidal behavior. Such information can help prioritize focused prevention strategies, as well as identify settings for prevention, early detection, and referral to services. The workplace provides an important site for suicide prevention activities because worksites have the structure and employee interaction to identify suicidality and to provide support and referral services. The objective of this analysis is to examine individual characteristics, suicide mechanism, and circumstances associated with work-related suicides. Work factors and other circumstances associated with suicides are from the in-depth investigation information in the National Violent Death Reporting System, which we included from 2013 to 2017.

## 2. Materials and Methods

### 2.1. Study Design and Data Source

This retrospective study of suicides examines data from the US National Violent Death Reporting System (NVDRS) from 2013 through 2017 (which was the latest year that data were available at the time of this analysis). Data were obtained through the approval process of the US Centers for Disease Control and Prevention (CDC)/National Center for Injury Prevention and Control (NCIPC) standard process. The study was approved by the University of Iowa Institutional Review Board.

NVDRS data are collected by states through a contract with CDC/NCIPC and include information from death certificates, autopsy reports, law enforcement investigation reports, and crime scene analyses. Between 2013 and 2017, the NVDRS system included 35 states as well as Puerto Rico and the District of Columbia. During these study years, 17 states reported data in all of the study years, and the remaining 18 states reported for at least one year. To assure comparisons were valid, we examined the proportion of suicides that were work-related and not work-related between the 17 states that reported each year to the proportions using all reporting states for each year. This comparison yielded similar results (Table 1). We used reports from all reporting states in the remainder of the analysis. Years prior to 2013 were excluded because the variables describing underlying circumstance were different in prior years.

In order to maximize representation of the sample to the working population, suicides within the age group of 16 (when most states issue work permits) to 65 (the most common age of retirement) were included. From 2013 to 2017, the NVDRS included 84,389 suicides in this age range from all states reporting to the system for at least one year.

### 2.2. Study Variables

Within the NVDRS, three variables identify if work has any relation to the decedent’s death. The death certificate indicates if the death was an “injury-at-work” with a yes/no response. According to instructions from the CDC [25], an injury at work applies to any occupation, not just the “usual occupation”, and includes deaths that occur during paid work, training, or volunteering in any area of a work premises, including while on a break, and including travel for business. The NVDRS codes a “job problem” if the record includes that the decedent experienced a problem related to work, such as the following: tensions with a co-worker or manager, poor performance review, increased pressure at work, fear of losing the job, or recently laid off from the job. If the record listed occupation as unemployed without any specific job problems identified, the death was not considered to have work factors. A “job crisis” was defined by the NVDRS as a job problem current at the time of death or had occurred within two weeks of the death (CDC 2020). Thus, the “job crisis” variable is a subset of the “job problem” variable for which the job problem was proximal in time and a priority factor identified in the suicide circumstances. The variables “job problem” and “job crisis” are coded by trained abstractors for each NVDRS site. A prior analysis has examined the frequency and overlap of these reporting sources [12].

Using these three NVDRS variables, we created three categories to indicate the extent to which the suicide was related to work. We coded work as a major factor in the suicide if the death certificate indicated the injury was at work or if the job problem was a crisis, a minor factor if the job problem variable was positive, but was not a crisis and the death certificate did not indicate injury at work, and not a factor if no variable identified any work or job ties. This three-category work factor variable was our primary dependent variable.

We examined demographic characteristics of suicide cases including sex, age, marital status, education, occupation, and race. Main independent variables were the mechanism of the suicide and the circumstances coded as being related to the suicide. The circumstances included a history of mental health problems, depressed mood, alcohol use problems, family or other relationship problems, a recent argument, a recent death, a history of suicide attempt, leaving a suicide note, physical health problems, financial problems, and eviction/loss of home. Definitions for circumstances and other NVDRS variables can be found in the NVDRS codebook [25].

### 2.3. Analysis

Suicides were categorized as a major factor, minor factor, or not a factor. The proportion of all suicides that were identified in each of the three categories were compared by year, sex, age, marriage, education, race, suicide mechanism, and circumstances using the Chi-Squared Test for Independence. Among the 84,389 suicides, 7515 (8.9%) did not have any recorded circumstances. Circumstances can be missing if the records from NVDRS data sources (medical examiner reports or law enforcement reports) were not available for abstraction, had insufficient detail to identify any circumstances, or had none of the abstracted circumstances associated with the death. We included all 84,389 cases to examine yearly trends and demographic characteristics. Only the 76,874 records with known circumstances were included to compare suicide mechanism and circumstances and for multivariable modeling.

To identify which circumstances related to a suicide were associated with work-related factors, we applied a stepwise model selection procedure based on baseline-category logit models. Suicides for which work was not a factor served as the reference. Two logit models were fitted to examine the work factor variable level of a major factor or a minor factor in comparison to the reference of not a factor. For example, let π1, π2, and π3 denote the proportion of a major factor, a minor factor, and not a factor respectively, and the two logit models compared with the reference of not a factor were: log(π1/π3) = X*β1, and log(π2/π3) = X*β2, in which the covariates (X) are the same in both models, but β1 and β2 are different coefficients. A significance level of 0.05 was required to allow a variable into the model and a significance level of 0.0001 was required for a variable to stay in the model. All statistical analyses were conducted using SAS 9.4 (SAS Institute, Cary, NC, USA) and R version 4.0.4 (The R Foundation, Vienna, Austria).

## 3. Results

Of the 84,389 suicides included in this analysis, more than 12.1% included some relatedness with work, of which 8.7% of the death investigations identified that work factors were a minor problem and 3.4% identified work factors as a major problem associated with the suicide (Table 1). The proportion of suicides that involved work factors did not change significantly from the period of 2013 to 2017. Trends were similar in the 17 states that reported data through the entire reporting period compared to the 37 states that reported for at least one of the study years.

Both minor and major work factors were more prevalent in suicides committed by males than females (Table 2). Work factors were the least prevalent among suicides in the age group of 16–20, which likely has the fewest full-time workers. Work factors were most common in suicides among those aged 35–54, with 9.9% involving work as a factor and 3.9% a major factor. Being married or divorced/separated had the highest prevalence of work factors contributing to their suicide, and those with a college education or above had the highest prevalence. Work factors were documented most frequently in suicides committed by those coded as being White, non-Hispanic.

Suicides among six occupations that identified 5% or more as involving work as a major factor included: management (6.8%), legal services (6.5%), protective services (5.6%), architecture and engineering (5.4%), life, physical, and social sciences (5.4%), and healthcare practitioners and technical (5.2%) (Table 2). The lowest were food preparation and service (2.9%), construction and extraction (2.7%), personal care and service (2.7%), healthcare support (2.6%), and community and social service (2.6%). The occupations that identified the highest percentage of work as a minor factor were legal services (16.6%), computer and mathematic services (14.2%), life, physical, and social sciences (13.8%), and business and financial operations (13.7%).

The 76,874 records (91.9% of all records) with known circumstances were included to compare suicide mechanism and circumstances (Table 3). The most common mechanisms of death among suicides that had identified work factors were hanging/asphyxiation (10% as a minor factor and 4.1% as a major factor) and firearms (10.6% as a minor factor and 3.7% as a major factor). The suicide circumstances more commonly associated with work factors were financial problems (33.6% as a minor factor and 7.5% as a major factor) and eviction or loss of home (22.9% as a minor factor and 5.4% as a major factor).

Table 4 presents effect estimates from a logistic model selecting variables that best predicted work factors (minor and major work factors combined). Overall, the model Concordance Index (also known as the c statistic) was 0.75, indicating a good prediction of work factors in suicide. Being male, between the ages of 21 and 54, and being married increased the likelihood of the suicide being work-related. Suicides among those aged 16 to 20 and above the age of 55 were less likely to be work-related. The odds of work-related suicide increased with increasing education levels. Poisoning as a mechanism of suicide was less likely to be work-related, with hanging and firearms being the most prevalent mechanisms among work-related suicides.

Eight occupations were associated with work-related suicide. Suicides among health practitioners had the highest odds of being work-related with an odds ratio of 1.82 (95% CI = 1.62–2.04), followed by management (Odds Ratio (OR)= 1.5; 95% Confidence Interval (CI) = 1.38–1.63). The odds of suicides being work-related were significantly elevated for business and financial operations (OR = 1.48; 95% CI = 1.28–1.70), protective services (OR = 1.44; 95% CI = 1.25–1.64), office and administrative support (OR = 1.36; 95% CI = 1.23–1.52), computer and mathematical occupations (OR = 1.32; 95% CI = 1.15–1.52), and sales and related occupations (OR = 1.28; 95% CI = 1.17–1.40). The odds of suicides among construction and extraction occupations being work-related were lower than other occupations (OR = 0.85; 95% CI = 0.79–0.92).

Among the ten NVDRS circumstances associated with work factors in suicide, the strongest association was with financial problems (OR = 4.7, 95% CI = 4.5–5.0), depressed mood (OR = 2.4, 95% CI = 2.3–2.5), and eviction/loss of home (OR = 1.6, 95% CI = 1.4–1.7). Other circumstances that were positively associated with work-related suicide were alcohol problems, relationship and family relationship problems, and leaving a suicide note. A history of suicide attempts, recent death of a loved one, and a recent argument were negatively associated with work-related suicide. Mental health problems (excluding depression) and physical health problems were not strongly associated with work-related suicide (not selected in the model).

## 4. Discussion

We found that suicides have more work-related circumstances than previously identified by a factor of up to ten-fold, with work identified as a major factor in more than 3% of suicides and a minor factor in approximately 9% [12]. Prior to the implementation of the National Violent Death Reporting System, data on specific work circumstances and other contributing factors were not widely available and were not available at a national level. This is the first study to use the NVDRS information from death investigations to identify the prevalence of work factors in suicides and the related circumstances.

Suicide cases that identified financial problems as related to the suicide were 4.7 times more likely, and those experiencing eviction/loss of home were 1.57 times more likely, to be identified as work-related. Prior studies have identified an association between financial strain and suicide/suicide attempts. For example, one prior study examined suicide circumstances among those aged 40 to 64 years old and found that job, financial, and legal problems increased from 32.9% to 37.5% from 2005 to 2010 [26]. Research using a national US sample found that financial strain, including unemployment, was associated with an increased odds of attempting suicide of approximately 1.5 [27]. A US policy evaluation found that a one dollar increase in the real minimum wage was associated with an average 1.9% decrease in the state suicide rate [28]. However, none of these studies examined specific circumstances that are related to work-related suicide.

We also found that depressed mood, alcohol problems, and relationship problems were associated with an increased odds of work-related suicide. These results are consistent with many studies that have found these circumstances associated with overall suicide risk [26,29,30,31,32]. Although a history of suicide attempt, death of a friend or family member, and prior argument have been associated with suicide risk [33,34,35], we found these circumstances to be negatively associated with work-relatedness of a suicide. The reason for these risk factors not being associated with work factors is not clear. Perhaps work-related factors that proceed a suicide are acute in nature, and the factors that make prior suicide history such a strong risk factor for a suicide are not frequently co-occurring with work factors.

Suicides among healthcare, management, business/financial operations, protective services, office/administrative support, computer/mathematical, and sales/related occupations were more likely to be work-related than all other occupations. Many of these occupations have been previously identified as being at high risk of suicide [18,19,20,21,22,23,24]. We found that suicides in the construction occupations were less likely to be work-related. Prior research, including estimates based on the NVDRS, have found suicide rates among construction workers to be among the highest of all occupations [20]. However, our findings are not contradictory, because Peterson et al. estimated suicide rates among the population by occupation, and we are estimating the odds that a suicide is work-related [20]. Thus, although suicide rates among construction workers are very high, these suicides are less likely to identify job problems as an underlying circumstance or have the death certificate indicate that the suicide was work-related. Our findings help identify occupations in which work-related factors are frequent among suicides, but do not identify occupations in which suicides are more prevalent. At the workplace level, work factors may be more prevalent in occupations that have inherently stressful work environments, either based on the work duties or work organization factors. Work factors could also be at the individual level, such as issues with work performance.

This study has several limitations. The NVDRS has recently expanded to all states but is not yet a population-based surveillance system. During the study years of 2013–2017, 15 states were not yet providing data and not all states were state-wide: 17 states reported for all 5 study years, and the number of reporting states grew from 17 to 37 over this period. Our sensitivity analysis found that trends in work factors did not vary substantially when using the 17 recurring states or all reporting states. Death investigations focus on the factors that are a priority for each individual case, and despite having clear definitions of work-related variables, not every investigation is going to include work factors, especially if they are not the main circumstances. Since death investigations and certifications are conducted by a large number of teams across each state, and these teams have various areas of expertise, there is considerable variance in how investigation information is collected. Variation likely also exists in state collection of NVDRS data. At the time of this analysis, data only through 2017 were available. With the many sources of data and the high-quality validation process at the federal level, there is a delay in access to data.

Despite these limitations, this study provides new information about the types of occupations and circumstances that are associated with work-related suicides. The workplace is an important site for suicide prevention because workplaces are a promising partner in the development and implementation of suicide prevention activities, and because workplaces have personnel, economic, and work culture impacts from suicide. Currently, workplaces have little information about the prevalence and co-occurrence of the underlying factors because little research has examined this topic.

## 5. Conclusions

Programs to prevent suicide can support the employee population by providing resources and expertise focused on the underlying circumstances contributing to the suicidal ideation, which include the circumstances identified in this analysis [36]. The evidence base for workplace suicide prevention is growing in some sectors such as law enforcement [37] and healthcare [38], but reviews consistently identify the inability of workplace prevention programs to focus on specific risk factors as a hindrance to program development and implementation [36,39,40]. Conceptually driven evaluations of outcomes are also lacking, in part because the specific short-term outcomes are difficult to define without information about underlying factors that can increase the risk for suicide. This study identified characteristics, occupations, and circumstances that are more likely to be associated with work-related than non-work-related suicides. Such information, which have not been previously reported, can help prioritize occupations and workers and can help focus prevention efforts. For example, these findings suggest that total worker health and wellness programs may consider incorporating financial support, mental health, alcohol reduction, and relationship health services into their programming to address circumstances associated with work-related suicide.

## Figures and Tables

**Table 1:** Proportion of suicides for which work was a minor, major, or no contributing factor, National Violent Death Reporting System, by all reporting states and 17 states reporting each year from 2013 through 2017.

Year	Number of States	Not a Factor N (%)	A Minor Factor N (%)	A Major Factor N (%)	Total Number of Suicides (N)
All Reporting States					
2013	17	9281 (86.13)	1137 (10.55)	357 (3.31)	10,775
2014	18	10,505 (87.21)	1094 (9.08)	447 (3.71)	12,046
2015	27	14,793 (88.34)	1431 (8.55)	522 (3.12)	16,746
2016	32	18,403 (87.95)	1822 (8.71)	700 (3.35)	20,925
2017	37	21,191 (88.68)	1860 (7.78)	846 (3.54)	23,897
Total	37	74,173 (87.89)	7344 (8.70)	2872 (3.40)	84,389
States Reporting for All Study Years					
2013	17	9281 (86.13)	1137 (10.55)	357 (3.31)	10,775
2014	17	9509 (86.92)	996 (9.10)	435 (3.98)	10,940
2015	17	10,075 (88.40)	922 (8.09)	400 (3.51)	11,397
2016	17	10,142 (87.63)	996 (8.61)	436 (3.77)	11,574
2017	17	10,660 (88.56)	898 (7.46)	479 (3.98)	12,037
Total	17	49,667 (87.56)	4949 (8.72)	2107 (3.71)	56,723

**Table 2:** Proportion of suicides for which work was a minor, major, or not a contributing factor, by demographic characteristics, National Violent Death Reporting System, 2013 through 2017.

Characteristic	Total Number	Not a Factor (%)	A Minor Factor (%)	A Major Factor (%)	Chi-Square Test
Overall Percent		87.89	8.70	3.40	
Sex ^1^					<0.0001
Male	64,466	86.59	9.60	3.82	
Female	19,922	92.12	5.81	2.07	
Age					<0.0001
16–20	5775	95.29	3.31	1.40	
21–34	23,884	89.20	7.75	3.05	
35–54	36,763	86.14	9.93	3.93	
55–64	17,967	87.37	9.19	3.44	
Marriage					<0.0001
Married/Civil Union/Widowed	26,606	85.92	9.54	4.54	
Never Married/Single	35,665	89.52	7.79	2.69	
Divorced/ Married but Separated	21,030	87.46	9.35	3.19	
Other/Unknown	1088	91.36	5.61	3.03	
Education					<0.0001
Less Than High School	11,292	92.84	5.16	1.99	
High School Diploma/Some College Credit, But No Degree	45,091	88.81	8.00	3.19	
College and Above	19,066	83.54	11.78	4.68	
Unknown	8940	86.31	10.15	3.55	
Race					<0.0001
White, Non-Hispanic	68,502	87.37	9.13	3.49	
Black or African American, Non-Hispanic	5669	90.40	6.70	2.89	
Hispanic	5564	90.31	6.52	3.16	
Other Race, Non-Hispanic	3370	89.38	7.42	3.20	
Other/Unknown	1284	90.26	7.32	2.41	
Occupation					<0.0001
Management	5089	80.80	12.42	6.78	
Legal	523	76.86	16.63	6.50	
Protective Service	1854	83.17	11.27	5.56	
Architecture and Engineering	1511	81.73	12.84	5.43	
Life, Physical, and Social Science	522	80.84	13.79	5.36	
Healthcare Practitioners and Technical	2736	82.57	12.21	5.23	
Business and Financial Operations	1663	81.36	13.71	4.93	
Computer and Mathematical	1646	81.35	14.16	4.50	
Sales and Related	5334	84.53	11.29	4.18	
Farming, Fishing, and Forestry	585	90.09	5.81	4.10	
Building and Grounds Cleaning and Maintenance	2573	88.26	7.73	4.00	
Educational Instruction and Library	1306	87.98	8.19	3.83	
Installation, Maintenance, and Repair	4367	86.42	9.78	3.80	
Transportation and Material Moving	6295	87.24	9.09	3.67	
Arts, Design, Entertainment, Sports, and Media	1737	86.36	10.07	3.57	
Production	5421	86.94	9.50	3.56	
Office and Administrative Support	3897	87.12	9.49	3.39	
Food Preparation and Serving Related	3145	89.63	7.47	2.89	
Personal Care and Service	1573	90.46	6.80	2.73	
Construction and Extraction	9337	89.38	7.98	2.65	
Community and Social Service	663	88.39	9.05	2.56	
Healthcare Support	1138	90.07	7.38	2.55	

^1^ One suicide was missing sex.

**Table 3:** Proportion of suicides for which work was a minor, major, or not a contributing factor, by mechanism and circumstances, National Violent Death Reporting System, 2013 through 2017 *.

Characteristic	Total Number	Not a Factor (%)	A Minor Factor (%)	A Major Factor (%)	Chi-Square Test
Overall percent		86.85	9.55	3.60	
Mechanism					<0.0001
Fall	1838	89.34	6.47	4.19	
Hanging/Strangulation/Suffocation	24,004	85.86	10.05	4.09	
Firearms	34,845	85.65	10.61	3.74	
Fire or Burns	330	90.91	6.36	2.73	
Drowning	734	90.46	6.81	2.72	
Poison	12,106	90.81	6.85	2.35	
Transportation	1261	91.99	5.87	2.14	
Other Weapon	1569	87.89	8.16	3.95	
Other/Unknown	185	90.27	5.95	3.78	
Circumstances					
Financial Problem	7661	58.86	33.61	7.53	<0.0001
Eviction or Loss of Home	3147	71.78	22.85	5.37	<0.0001
Depressed Mood	28,059	79.00	15.96	5.04	<0.0001
Relationship Problems with Others	1892	80.81	14.43	4.76	<0.0001
Alcohol Problem	15,621	82.84	13.17	3.98	<0.0001
Suicide Note	25,891	85.66	10.45	3.90	<0.0001
Family Relationship Problem	7581	83.38	13.03	3.59	<0.0001
Mental Health Problem	38,902	86.97	9.78	3.25	<0.0001
Death Friend or Family, or Other	4318	86.85	9.55	3.60	0.0228
Physical Health Problem	11,695	85.75	11.21	3.04	<0.0001
Argument	13,732	88.68	8.60	2.72	<0.0001
Suicide Attempt History	16,915	88.46	8.95	2.59	<0.0001

* Note: 7515 subjects with unknown circumstances were excluded from this table and from multivariable models.

**Table 4:** Characteristics and circumstances predicting work-related suicides (minor and major factors combined), National Violent Death Reporting System, 2013 through 2017 *.

Characteristic/Circumstance	Total Number	Odds Ratio	95% CI Lower Limits	95% CI Upper Limits
Demographic Characteristics				
Male	58,346	1.96	1.84	2.09
Age group 16 to 20	5209	0.52	0.45	0.59
Age group 55 to 64	16,396	0.81	0.76	0.86
Married or Civil Union, or Widowed	24,307	1.26	1.20	1.32
Education Less Than High School	10,139	0.74	0.68	0.80
Education College and Above	17,533	1.34	1.27	1.41
Mechanism of Poison	12,105	0.73	0.67	0.78
Occupation				
Occupation: Healthcare Practitioners	2557	1.82	1.62	2.04
Occupation: Management	4670	1.50	1.38	1.63
Occupation: Business and Financial Operations	1538	1.48	1.28	1.70
Occupation: Protective Service	1688	1.44	1.25	1.64
Occupation: Office and Administrative Support	3617	1.36	1.23	1.52
Occupation: Computer and Mathematical	1522	1.32	1.15	1.52
Occupation: Sales and Related Occupations	4916	1.28	1.17	1.40
Occupation: Construction and Extraction	8496	0.85	0.79	0.92
Circumstances				
Financial Problem	7661	4.70	4.45	4.97
Depressed Mood	28,060	2.39	2.29	2.50
Eviction or Loss of Home	3147	1.57	1.43	1.72
Alcohol Problem	15,621	1.32	1.25	1.39
Relationship Problem with Others	1892	1.36	1.19	1.54
Family Relationship	7581	1.25	1.17	1.35
Suicide Note	25,892	1.12	1.06	1.16
Suicide Attempt History	16,915	0.87	0.82	0.92
Death Friend or Family, or Other	4318	0.83	0.75	0.91
Argument	13,732	0.79	0.74	0.84

* Note: 7515 subjects with unknown circumstances were excluded.

## Data Availability

Access to these data are through the Centers for Disease Control and Prevention/National Center for Injury Prevention and Control: https://www.cdc.gov/violenceprevention/datasources/nvdrs/index.html.
